# Endophenotypic effects of the *SORL1* variant rs2298813 on regional brain volume in patients with late-onset Alzheimer’s disease

**DOI:** 10.3389/fnagi.2022.885090

**Published:** 2022-08-05

**Authors:** Chun-Yu Chen, Yung-Shuan Lin, Wei-Ju Lee, Yi-Chu Liao, Yu-Shan Kuo, Albert C. Yang, Jong-Ling Fuh

**Affiliations:** ^1^Department of Medicine, Taipei Veterans General Hospital Yuli Branch, Hualien, Taiwan; ^2^Division of General Neurology, Neurological Institute, Taipei Veterans General Hospital, Taipei, Taiwan; ^3^Faculty of Medicine, National Yang Ming Chiao Tung University, Taipei, Taiwan; ^4^Brain Research Center, National Yang-Ming Chiao Tung University, Taipei, Taiwan; ^5^Institute of Brain Science, National Yang Ming Chiao Tung University, Taipei, Taiwan; ^6^Neurological Institute, Taichung Veterans General Hospital, Taichung, Taiwan; ^7^Dementia Center and Center for Geriatrics and Gerontology, Taichung Veterans General Hospital, Taichung, Taiwan; ^8^Department of Post-Baccalaureate Medicine, College of Medicine, National Chung Hsing University, Taichung, Taiwan; ^9^Division of Peripheral Neurology, Neurological Institute, Taipei Veterans General Hospital, Taipei, Taiwan

**Keywords:** Alzheimer’s disease, dementia, sortilin-related receptor 1 gene, SORL1, MRI

## Abstract

**Introduction**: Two common variants of sortilin-related receptor 1 gene (*SORL1*), rs2298813 and rs1784933, have been associated with late-onset Alzheimer’s disease (AD) in the Han Chinese population in Taiwan. However, neuroimaging correlates of these two *SORL1* variants remain unknown. We aimed to determine whether the two *SORL1* polymorphisms were associated with any volumetric differences in brain regions in late-onset AD patients.

**Methods**: We recruited 200 patients with late-onset AD from Taipei Veterans General Hospital. All patients received a structural magnetic resonance (MR) imaging brain scan and completed a battery of neurocognitive tests at enrollment. We followed up to assess changes in Mini-Mental State Examination (MMSE) scores in 155 patients (77.5%) at an interval of 2 years. Volumetric measures and cortical thickness of various brain regions were performed using FreeSurfer. Regression analysis controlled for apolipoprotein E status. Multiple comparisons were corrected for using the false discovery rate.

**Results**: The homozygous major allele of rs2298813 was associated with larger volumes in the right putamen (*p* = 0.0442) and right pallidum (*p* = 0.0346). There was no link between the rs1784933 genotypes with any regional volume or thickness of the brain. In the rs2298813 homozygous major allele carriers, the right putaminal volume was associated with verbal fluency (*p* = 0.008), and both the right putaminal and pallidal volumes were predictive of clinical progression at follow-up (*p* = 0.020). In the minor allele carriers, neither of the nuclei was related to cognitive test performance or clinical progression.

**Conclusion**: The major and minor alleles of rs2298813 had differential effects on the right lentiform nucleus volume and distinctively modulated the association between the regional volume and cognitive function in patients with AD.

## Introduction

Accumulation of the neurotoxic proteolytic derivative of amyloid beta precursor protein (APP), amyloid-beta (Aβ) peptide, is proposed to be key to the pathogenesis of Alzheimer’s disease (AD) (Hardy and Selkoe, 2002). Sortilin-related receptor 1 (*SORL1*) encodes a mosaic protein (SORLA) consisting of several distinct domains, including the vacuolar protein sorting 10 protein (VPS10P) domain for Aβ binding and the low-density lipoprotein receptor domain for APP binding and lipoprotein binding (Barthelson et al., 2020). SORLA acts as a sorting receptor for retrograde trafficking of APP to the trans-Golgi-network to prevent APP processing to Aβ and anterograde movement of Aβ for lysosomal degradation (Andersen et al., 2016). *SORL1* mutations in human neurons lead to reduced levels of SORLA, resulting in defects of the neuronal endolysosome function and autophagy (Hung et al., 2021). In addition, being a low-density lipoprotein receptor, SORLA mediates neuronal uptake of apolipoprotein E (ApoE)-rich lipoproteins (Yajima et al., 2015), the misfolding of which contributes significantly to AD pathogenesis (Barthelson et al., 2020). Ablation of SORLA expression increases Aβ in the brain of knockout mice (Andersen et al., 2005), and SORLA-overexpressing cells have remarkably reduced levels of extracellular Aβ and lower levels of intracellular APP (Offe et al., 2006). In the brains of AD patients, *SORL1* expression is reduced (Scherzer et al., 2004). Moreover, both common and rare variants of *SORL1* have been associated with late-onset and early-onset AD, respectively (Campion et al., 2019).

*SORL1* variants were first identified, among several genes belonging to endocytic pathways, to be associated with sporadic AD by a pioneering study in Caucasians (Rogaeva et al., 2007). Targeted single-nucleotide polymorphism (SNP) analyses and genome-wide association studies have validated the association not only in populations of Caucasian origin but also in Asian populations (Reitz et al., 2011; Lambert et al., 2013; Miyashita et al., 2013). A number of studies have shown significant associations between specific *SORL1* polymorphisms and various phenotypes in AD patients, including lower Aβ levels in cerebrospinal fluid (Alexopoulos et al., 2011) and serum (Chou et al., 2016), increased tau protein in cerebrospinal fluid (Louwersheimer et al., 2015), hippocampal atrophy (Cuenco et al., 2008; Louwersheimer et al., 2015; Xiromerisiou et al., 2021), white matter hyperintensity (Cuenco et al., 2008), frontal symptoms (Huang et al., 2020), rate of cognitive decline (Hsieh et al., 2021), and Parkinsonian features (Cuccaro et al., 2016; Xiromerisiou et al., 2021).

We have previously reported that in the Han Chinese population in Taiwan, two common variants of *SORL1*, rs2298813, and rs1784933, were associated with late-onset AD (Chou et al., 2016). In the elderly population in Australia and the United States, rs2298813 has also been identified in individuals with late-onset AD (Assareh et al., 2014; Cuccaro et al., 2016). The association of rs1784933 with the risk of late-onset AD has also been reported in the Han Chinese population in China (Feng et al., 2015; Zhang et al., 2017) and Mexicans (Toral-Rios et al., 2022). The SNPrs2298813 is located at the VPS10P domain, and the nonsynonymous substitution of alanine to threonine at the 528th residue (A528T) of SORLA has been shown to increase the secretion of Aβ42, soluble APPα, and APPβ *in vitro* (Vardarajan et al., 2015). The SNPrs1784933 is located in the 3’ region of *SORL1*, and minor allele carriers with late-onset AD had lower plasma concentrations of Aβ42 (Chou et al., 2016). The endophenotypic effects of various *SORL1* polymorphisms on the brain have been revealed in nondemented individuals (Liang et al., 2015; Huang et al., 2016; Yin et al., 2016; Li et al., 2017). For example, among the non-demented elders, rs1699102 was associated with gray matter volume of the right middle temporal pole (Li et al., 2017), and rs1784933 and rs753780 was associated with right parahippocampal volume (Yin et al., 2016). However, the neuroimaging correlates of rs2298813 and rs1784933 have not been established in AD patients. We, therefore, aimed to examine the associations between the two *SORL1* SNPs and gray matter volume and cortical thickness of different brain regions in late-onset AD patients.

## Materials and Methods

### Subjects

A total of 200 patients with late-onset AD were enrolled from Taipei Veterans General Hospital, Taiwan. All participants were of Han Chinese descent in Taiwan. Probable AD was diagnosed based on the criteria of the National Institute of Neurological and Communicative Disorders and Stroke/Alzheimer’s Disease and Related Disorders Association (Mckhann et al., 2011). The diagnostic survey included history queries (including confirmation of ethnicity by family history), neurological examinations, laboratory tests (including thyroid function, vitamin B12, folate, treponemal tests, renal function, liver enzymes, electrolytes, cell counts, glucose, etc.), and magnetic resonance (MR) imaging of the brain. A subset of patients (*n* = 92) was screened for cognitive fluctuation using the Mayo fluctuation scale (Ferman et al., 2004). The study was approved by the institutional review boards of Taipei Veterans General Hospital. Informed consent was obtained from all patients in accordance with our institutional guidelines and the recommendations of the Declaration of Helsinki.

### Genotyping

Whole blood genomic DNA was extracted with a commercial kit in accordance with the manufacturer’s instructions (QIAGEN, Hilden, Germany). The alleles of APOE (ε2, ε3, and ε4) were determined by rs429358 and rs7412 (Chen et al., 2012). Genotyping of the two *SORL1* SNPs (rs2298813 and rs1784933) and APOE alleles was performed using the TaqMan genotyping assay (Applied Biosystems, Foster City, CA, USA). Polymerase chain reactions were carried out in 96-well microplates using an ABI 7500 real-time polymerase chain reaction system (Applied Biosystems International, Framingham, MA). For allele discrimination, the fluorescence signal from the TaqMan polymerase chain reaction was analyzed using SDS software version 1.2.3 (Applied Biosystems International, Framingham, MA). Duplicate confirmation was performed if the initial genotyping result was undetermined. The failure rate for rs1784933 and rs2298813 was 0.59% and 1.19% respectively.

### Cognitive testing

Global cognitive performance was assessed in each patient using the Mini-Mental State Examination (MMSE; Folstein et al., 1975). Cognitive domain-specific tests were performed on all patients. Attention was tested by the forward and backward digit span tests from the Wechsler Memory Scale-IV (Wechsler, 2009), memory by the 12-item word recall test (Vanderploeg et al., 2000), language and executive function by the verbal fluency category test (Harrison et al., 2000), processing speed by the Trail Making Test A (Lu and Bigler, 2002), and naming by the Boston naming test (Mack et al., 1992). We followed up to assess changes in MMSE scores over a mean interval of approximately 2 years in these patients. Rapid clinical progression was defined as a decrease in follow-up MMSE by at least 3 points per year (Schmidt et al., 2011).

### Imaging analysis

MR images were scanned at Taipei Veterans General Hospital, Taipei, Taiwan, on a 3.0-T GE Signa MRI scanner (GE Medical Systems, Milwaukee, WI, USA). High-resolution anatomic MR images were acquired through a 3D inversion-recovery fast spoiled gradient-echo (BRAVO) sequence. The high-resolution structural T1 images were processed using FreeSurfer version 5.3^1^ based on the 2010 Desikan-Killiany atlas. Cortical reconstruction using FreeSurfer involved automated and manual processing. The automated processing included motion correction, nonbrain tissue removal (Fischl et al., 2002), Talairach transformation, segmentation of the subcortical white matter and deep gray matter structures, intensity normalization, tessellation of the boundary between gray and white matter (Segonne et al., 2007), automated topology correction, and surface deformation. When necessary, manual editing was undertaken to correct the pial surface error, skull strip error, or intensity normalization error following the FreeSurfer tutorial. Cortical thickness was calculated as the distance between the white and gray matter surfaces at each point across the regional cortex. There were 68 regions of cortical thickness and 21 regions of gray matter volume included in the statistical analysis. AD-related brain regions included volumes in the hippocampus and thickness of the parahippocampal gyrus, posterior cingulate cortex, middle temporal gyrus, and entorhinal cortex (Yin et al., 2016). Other regions (other than AD-related regions) included all regions except the AD-related brain regions (19 regions of gray matter volume and 60 regions of cortical thickness).

### Statistical analysis

Hardy-Weinberg equilibrium tests were conducted for each SNP. A dominant model of inheritance of the minor allele was presumed to test the associations between *SORL1* SNPs and imaging parameters. The analyses were executed with PASW Statistics software (version 25.0; SPSS, Chicago, IL, USA). Data are expressed as the mean ± standard deviation or number of patients (%), as appropriate. The χ^2^ test was performed for categorical variables, and the t-test was performed for the comparion of two means. Multivariate linear regression analyses were used to assess the relationships between regional cortical thickness or gray matter volume and the *SORL1* SNPs or cognitive test results. The covariates included age, gender, education level, and APOE status. A logistic regression model was conducted to investigate the associations between rapid clinical progression or MMSE changes (points/year) at follow-up and features of selected brain regions with covariates that included age, gender, education level, and APOE status. For regression analysis involving gray matter volume, the estimated intracranial volume was additionally included as a covariate. Multiple comparisons were corrected with the false discovery rate (Benjamini–Hochberg procedure) respectively for gray matter volume (21 regions) and cortical thickness (70 regions). Statistical significance was taken at *P* < 0.05 or Benjamini–Hochberg corrected *P*_c_ < 0.05.

## Results

### Demographic data

The demographic data for those with rs2298813 and rs1784933 are shown in Table 1. Regarding rs2298813, there were 147 patients carrying the wild homozygote (GG), and 51 patients carrying the minor allele (51 AG and 3 AA). Regarding rs1784933, there were 92 patients carrying the wild homozygote (AA), and 109 patients carrying the minor allele (93 AG and 16 GG). Age, gender, MMSE score, years of education, disease duration, and APOE status did not differ between the homozygous major allele carriers and the minor allele carriers of both SNPs. The Mayo fluctuation scale scores tended to be higher in minor allele carriers than in homozygous major allele carriers of rs2298813 (*p* = 0.052) but were similar between minor allele carriers and non carriers of rs1784933 (*p* = 0.935). For both SNPs, the minor allele carriers performed similarly in all the neuropsychiatric tests as did the homozygous major allele carriers (Table 1). A total of 155 patients (77.5%) had a follow-up MMSE assessment after a mean interval of 2.1 ± 0.8 years, and 37 patients had clinical progression. At follow-up, the minor allele carriers of both SNPs showed no differences in the risk of clinical progression vs. the homozygous major allele carriers.

**Table 1:** The demographic data and cognitive test performance of the homozygous major allele carriers and minor allele carriers.

	Homozygous major allele *n* = 146)	Minor allele (*n* = 54)	*P*
**rs2298813**
*Demographic*
Age, years	77.5 ± 7.9	78.2 ± 6.7	0.590
Gender (male)	67 (45.9%)	23 (42.6%)	0.677
Education level, years	10.0 ± 4.2	9.1 ± 5.0	0.240
Disease duration, months	31.8 ± 42.5	32.9 ± 29.4	0.860
Mayo fluctuations scale	1.2 ± 1.3	1.8 ± 1.1	0.052
Clinical progression	26/118 (22.0%)	10/38 (26.3)	0.586
*Genetic test*
APOE ε4	41 (28.1%)	24 (44.4%)	0.074
ε4/ε4	4 (2.7%)	2 (3.7%)
*Cognitive test*
MMSE score	19.9 ± 5.1	20.9 ± 5.2	0.197
12-item word recall	1.5 ± 2.0	2.1 ± 2.5	0.127
Forward digit span	8.7 ± 2.9	8.8 ± 2.7	0.900
Backward digit span	4.2 ± 1.8	4.6 ± 2.4	0.290
Verbal fluency	7.1 ± 2.9	7.9 ± 2.9	0.079
Boston Naming	11.1 ± 2.5	11.2 ± 2.6	0.808
Trail Making, seconds	146.4 ± 97.4	128.9 ± 81.0	0.261
**rs1784933**	(n = 92)	(n = 108)	
*Demographic*
Age (years)	77.7 ± 8.2	77.55 ± 7.38	0.870
Gender (male)	45 (48.9%)	45 (41.7%)	0.305
Education (years)	9.4 ± 4.2	10.0 ± 4.5	0.341
Disease duration (months)	27.6 ± 25.0	36.1 ± 48.0	0.111
Mayo fluctuations scale	1.4 ± 1.3	1.4 ± 1.2	0.935
Clinical progression	19/67 (28.4%)	18/88 (20.5%)	0.253
*Genetic test*
APOE ε4	29 (31.5%)	36 (33.3%)	0.770
ε4/ε4	2 (2.2%)	4 (3.7%)
*Cognitive test*
MMSE	19.8 ± 5.4	20.5 ± 4.7	0.313
12-item word recall	1.7 ± 2.1	1.7 ± 2.3	0.983
Forward digit span	8.5 ± 2.9	8.9 ± 2.8	0.253
Backward digit span	4.2 ± 1.9	4.4 ± 2.0	0.415
Verbal fluency	7.2 ± 3.1	7.4 ± 2.8	0.696
Boston Naming	11.0 ± 2.7	11.3 ± 2.4	0.367
Trail Making, seconds	145.7 ± 96.1	140.7 ± 93.7	0.715

*APOE, apolipoprotein E; MMSE, Mini-Mental State Examination*.

### Associations of rs2298813 and rs1784933 with regional cortical thickness and gray matter volume

**Figure 1:**
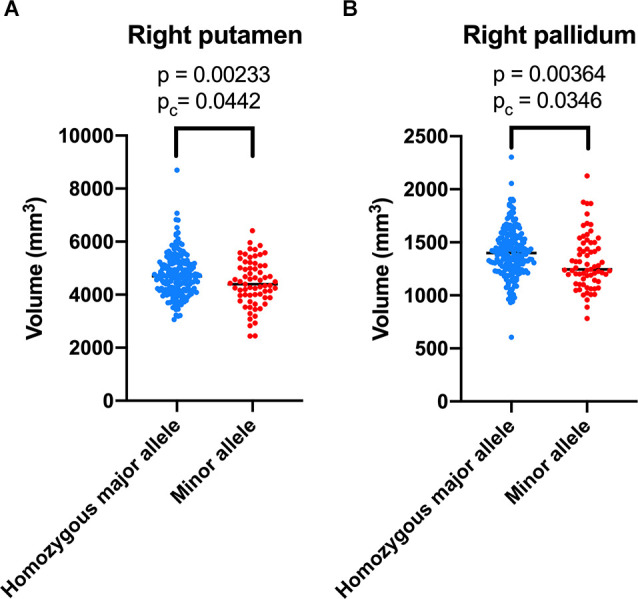
Volumetric comparison of the right putamen **(A)** and right pallidum **(B)** between the homozygous major allele carriers and minor allele carriers of rs2298813.

The correlations of the genotype of rs2298813 and rs1784933 with regional gray matter volumes or regional cortical thickness in AD-related brain regions and other brain regions are shown in Tables s 2, 3, respectively. The genotype of rs2298813 was not associated with any of the AD-related brain regions (2). Among other brain regions, there were significant partial correlations of volumes in the right putamen and right pallidum with the genotype of rs2298813; the homozygous major allele was associated with larger volumes in the two regions (Figure 1, Table 3). With respect to rs1784933, there was no association with any of the brain regions.

**Table 2:** Associations of homozygous major allele carriers of rs2298813 and rs1784933 with regional cortical thickness/gray matter volume in Alzheimer’s disease (AD)-related brain regions.

rs2298813			rs1784933		
AD-related brain regions	*r*	*p*	*p*_c_	*r*	*p*	*p*_c_
Gray matter volume						
Right hippocampus	0.062	0.392	0.784	0.096	0.181	0.362
Left hippocampus	0.056	0.441	0.441	0.069	0.338	0.676
Cortical thickness						
Right parahippocampal gyrus	0.122	0.0874	0.350	−0.007	0.921	0.921
Left parahippocampal gyrus	0.162	0.0231	0.185	−0.040	0.574	0.765
Right posterior cingulate gyrus	0.047	0.517	1.000	0.087	0.224	0.896
Left posterior cingulate gyrus	−0.040	0.582	0.931	−0.051	0.478	0.765
Right middle temporal gyrus	−0.080	0.263	0.701	−0.121	0.0898	0.718
Left middle temporal gyrus	−0.032	0.652	0.869	−0.073	0.311	0.622
Right entorhinal cortex	0.017	0.810	0.926	−0.074	0.301	0.803
Left entorhinal cortex	0.016	0.820	0.820	−0.030	0.681	0.778

*r, partial correlation coefficient*.

**Table 3:** Associations of homozygous major allele carriers of rs2298813 and rs1784933 with regional cortical thickness/gray matter volume in other brain regions.

rs2298813	*r*	*p*	*p*_c_
**Gray matter volume**
Right putamen	−0.217	0.00233	0.0442
Right pallidum	−0.207	0.00364	0.0346
rs1784933	*r*	*p*	*p*_c_
**Cortical thickness**
Right superior temporal gyrus	−0.171	0.0163	0.978
Right postcentral gyrus	−0.154	0.0307	0.921
Right pars triangularis	−0.149	0.0370	0.740
Left precentral gyrus	−0.144	0.0444	0.666

*r, partial correlation coefficient*.

### Associations of the right lentiform nucleus with cognitive test performance in the homozygous major allele carriers and minor allele carriers of rs2298813

The associations between the right lentiform nucleus and cognitive test performance for the homozygous major allele carriers and minor allele carriers of rs2298813 are shown in Table 4. Among the homozygous major allele carriers, the right putaminal volume was significantly related to verbal fluency (*p* = 008). At follow-up, the volumes in the right putamen and right pallidum at baseline were positively related to the change in MMSE scores (partial *r* = 0.181, *p* = 0.056 for the right putamen; partial *r* = 0.192, *p* = 0.042 for the right pallidum, Table 5). Logistic regression revealed that lower volumes in both the right putamen (*p* = 0.020) and the right pallidum (*p* = 0.013) were predictive of clinical progression at follow-up (Table 5).

Among the minor allele carriers, the volumes of the right putamen and the right pallidum were not associated with any of the cognitive test performance (Table 4). At follow-up, neither the right putaminal volume nor the right pallidal volume was associated with the change in MMSE scores (partial *r* = 0.043, *p* = 0.814 for the right putamen; partial *r* = -0.138, *p* = 0.444 for the right pallidum) or clinical progression (*p* = 0.791 for the right putamen, *p* = 0.191 for the right pallidum, Table 5).

**Table 4:** Associations of the volume of the right lentiform nucleus with cognitive test performance in homozygous major allele carriers (*n* = 146) and minor allele carriers (*n* = 54) of rs2298813.

Homozygous major allele				Minor allele			
	Right putamen		Right pallidum		Right putamen		Right pallidum	
	r	p	r	p	r	p	r	p
MMSE	0.161	0.056	0.061	0.473	0.151	0.302	0.131	0.371
12-item word recall	0.088	0.299	0.020	0.815	−0.149	0.311	−0.093	0.529
Forward digit span	0.121	0.152	−0.030	0.723	0.239	0.098	0.223	0.123
Backward digit span	0.013	0.878	0.016	0.847	0.098	0.501	0.186	0.201
Verbal fluency	0.224	0.008	0.058	0.494	0.037	0.802	0.057	0.696
Boston Naming	0.100	0.238	0.092	0.276	0.159	0.276	0.209	0.149
Trail Making	−0.146	0.090	0.009	0.916	−0.121	0.435	−0.086	0.578

*MMSE, Mini-Mental State Examination; r, partial correlation coefficient*.

**Table 5:** Associations of the volumes of th right lentiform nucleus at baseline with clinical progression and MMSE changes at follow-up in homozygous major allele carriers (*n* = 118) and minor allele carriers (*n* = 38) of rs2298813.

Homozygous major allele		Minor allele	
Clinical progression	OR (95% CI)	*p*	OR (95% CI)	*p*
Right putaminal volume (mm^3^)	0.999 (0.998–1.000)	0.020	1.000 (0.999–1.001)	0.791
Right pallidal volume (mm^3^)	0.997 (0.994–0.999)	0.013	1.003 (0.998–1.009)	0.191
MMSE changes at follow-up	Standardized β	*p*	Standardized β	*p*
Right putaminal volume (mm^3^)	0.188	0.056	−0.041	0.814
Right pallidal volume (mm^3^)	0.211	0.042	−0.179	0.444

*CI, confidence interval; MMSE, Mini-Mental State Examination; OR, odds ratio*.

## Discussion

We found that the volume of the right lentiform nucleus differed between the homozygous major allele carriers of rs2298813 and the minor allele carriers in late-onset AD patients. With respect to rs1784933, there were no neuroimaging correlates of the genotype. Among the homozygous major allele carriers of rs2298813, there was an association between right putaminal volume and verbal fluency performance, and the right putaminal and right pallidal volumes were predictive of clinical progression. On the contrary, the minor allele carriers of rs2298813 had a smaller volume of the right lentiform nucleus and a higher cognitive fluctuation score. However, the right putaminal and right pallidal volumes were not related to neurocognitive test performance nor predictive of clinical progression among these patients. It appeared that the major and minor alleles of rs2298813 had differential effects on the volume of the right lentiform nucleus and differentially modulated the association between the right lentiform nucleus and cognitive function.

The earliest associations between rs2298813 and rs1784933 and brain atrophy were delineated in a collection of autopsied AD brains (Cuenco et al., 2008). The 3-SNP haplotypes containing the rs1784933 region were related to pathological scoring and MRI traits of hippocampal atrophy in white AD patients, whereas no significant associations were identified for rs2298813 (Cuenco et al., 2008). In an Australian cohort, a 3-SNP haplotype containing rs2298813 was associated with whole brain atrophy in both males and females, but rs2298813 was not individually associated with brain atrophy (Assareh et al., 2014). A study using the Alzheimer’s Disease Neuroimaging Initiative database investigated the effect of eight *SORL1* SNPs, including rs2298813 and rs1784933, on AD-related brain atrophy in subjects with normal cognition or mild cognitive impairment (MCI) (Yin et al., 2016). The A allele of rs2298813 tended to be associated with a lower volume in the right parahippocampal gyrus, and the G allele of rs1784933 was found to be associated with a higher rate of atrophy in the right parahippocampal gyrus across a 2-year span (Yin et al., 2016). In a young healthy Caucasian population, 117SNPs in and surrounding *SORL1* were examined to determine any association with hippocampal volume, and the majority of significant associations occurred at the 3’ region, where rs1784933 is located (Bralten et al., 2011). The discrepancy in the relationship between rs2298813/rs1784933 and brain atrophy may lie in ethnic differences (Jin et al., 2013) and the small effect of a single SNP that may be better demonstrated in quantitative measurement of brain volume or in haplotype analysis.

The putamen and pallidum (forming the lentiform nucleus) are essential elements of the extrapyramidal system and are usually involved in motor disturbances, such as Parkinsonism or Huntington’s disease (Albin et al., 1989). Although not included among the typical AD-related brain structures, numerous studies have disclosed an association of these regions with AD. The volume of the putamen has been shown to be reduced in AD brains relative to subjects with MCI or normal aging (De Jong et al., 2008; Roh et al., 2011; Cho et al., 2014; Tang et al., 2014; Eustache et al., 2016; Pini et al., 2016). Such reductions can occur as early as in the prodromal stage (Eustache et al., 2016). Atrophy in the pallidum has not been consistently observed in previous studies (Cho et al., 2014; Pini et al., 2016). The pallidum is relatively resistant to degeneration even in moderate stages of the disease (Roh et al., 2011) although mild atrophy may be observed (Li et al., 2013; Tang et al., 2014; Wang et al., 2018). AD pathology has been demonstrated to deposit heavily in the putamen and less in the pallidum (Braak and Braak, 1990). Similarly, iron detection using MR techniques (quantitative susceptibility mapping or phase imaging) has revealed iron accumulation in the putamen and pallidum (Bartzokis et al., 2000; Cogswell et al., 2021), which was associated with higher amyloid PET standardized uptake value ratios (Cogswell et al., 2021). The association of putaminal or pallidal atrophy with common variants of *SORL1* has not been previously specified in AD patients. The most relevant study was conducted by Huang et al. investigating the effect of rs3824968 on gray matter volume in a nondemented Chinese population across a wide age span (Huang et al., 2016). Participants carrying the A allele had accelerated atrophy with age in the right putamen (Huang et al., 2016). Thus, we are the first to identify a link between putaminal/pallidal atrophy and *SORL1* polymorphisms in AD.

The putamen and pallidum also have functions related to cognition. In patients with Parkinson’s disease, deep brain stimulation of the pallidum has been shown to improve verbal fluency. (Lee et al., 2018) In human immunodeficiency virus-associated neurocognitive impairment, there was an association between cognitive impairment and putaminal volume (Qi et al., 2021). Among patients with behavioral variant frontotemporal dementia, there is a relationship between atrophy in the putamen and the theory of mind impairment (Baez et al., 2019). In support of this, in our study, relationships between both verbal fluency and the annual rate of changes in MMSE scores and right putaminal volume were observed in the homozygous major allele carriers of rs2298813. In contrast, there were no significant associations between right putaminal/right pallidal volumes and cognitive test performance or clinical progression in the minor allele carriers even though the right lentiform nucleus was smaller in these patients. This may suggest that in this subgroup of patients, atrophy in the right lentiform nucleus did not have a deleterious impact on cognitive function. The atrophy may, for example, have more effect on motor function. Further study is needed to elucidate the phenotypic effect of regional atrophy in the right lentiform nucleus in the minor allele carriers of rs2298813.

The link between rs2298813 and the lentiform nucleus volume may provide a pathophysiological basis for the association between AD and parkinsonism. Indeed, Parkinsonism is not uncommon in AD. Extrapyramidal signs can be detected in one-third of AD patients during the course of the disease (Scarmeas et al., 2004), and Parkinsonian features are related to neuronal loss in the substantia nigra and putamen (Horvath et al., 2014). A previous study reported that three out of four patients with late-onset AD with Parkinsonism carried the A allele of rs2298813 (Cuccaro et al., 2016). In a case report, 4 AD patients with Parkinsonism and psychiatric symptoms were found to have novel mutations in *SORL1*, with two mutations at the VPS10P region, where rs2298813 is located (Qiu et al., 2021). Our minor allele carriers also had higher scores on the Mayo fluctuation scale, implying a link with features of Lewy body dementia. Moreover, the minor allele of rs2298813 has been found to increase the risk of developing dementia in patients with Parkinson’s disease (Maple-Grodem et al., 2018). In the northern Chinese population, rs2298813 was associated with an increased risk of Parkinson’s disease (Wang et al., 2022). In addition to its involvement in the APP pathway, SORLA also mediates the trophic pathway involving glial cell line-derived neurotrophic factor (Glerup et al., 2013), the absence of which could lead to the loss of dopaminergic neurons (Lin et al., 1993).

There were several limitations of the current study. First, we did not systemically qualify and quantify the motor symptoms in these patients. Therefore, the postulated relationship between atrophy in the putamen and pallidum and pyramidal/extrapyramidal symptoms warrants further study. Second, these patients were followed up for an average of 2 years, so a longer follow-up duration may better confirm the cognitive or motor effects of the volumetric changes in the putamen and pallidum. Third, the sample size is much smaller in the minor allele carrier of rs2298813, so the absence of an association with cognitive test performance may be a consequence of low power. The inclusion of more patients with this genotype would help to confirm our findings.

## Conclusion

The volume of the right lentiform nucleus was associated with cognitive function and clinical progression in late-onset AD patients with the homozygous major allele of rs2298813. Whereas, among the minor allele carriers, the volume of the right lentiform nucleus was smaller, and was not associated with cognitive function or clinical progression.

## Data Availability Statement

The original contributions presented in the study are included in the article, further inquiries can be directed to the corresponding author.

## Ethics Statement

The studies involving human participants were reviewed and approved by the institutional review boards of Taipei Veterans General Hospital (IRB number 2012-05-033B). The patients/participants provided their written informed consent to participate in this study.

## Author Contributions

C-YC wrote the manuscript. J-LF, C-YC, Y-CL, and W-JL contributed to the study concept and design. C-YC, Y-SL, W-JL, AY, and J-LF contributed to analysis and interpretation of data. Y-SL, W-JL, Y-SK, and J-LF contributed to acquisition of data. W-JL, Y-CL, and J-LF revised the manuscript. All authors contributed to the article and approved the submitted version.

## Funding

This study was supported by grants from the National Health Research Institutes, Taiwan (NHRI-11A1-CG-CO-05-2225-1), the Ministry of Science and Technology, Taiwan (MOST 109-2314-B-075-052-MY2, 110-2321-B-001-011-, 110-2321-B-A49A-502-, 110-2634-F-A49-005-), Taipei Veterans General Hospital (V110C-057, VGHUST110-G1-5-1, V111C-216) and the Brain Research Center, National Yang Ming Chiao Tung University from the Featured Areas Research Center Program within the framework of the Higher Education Sprout Project by the Ministry of Education (MOE) in Taiwan.

## Footnotes

^1^ http://surfer.nmr.mgh.harvard.edu/

## ABBREVIATIONS

Aβ: amyloid-beta
AD: Alzheimer’s disease
APOE: apolipoprotein E
APP: amyloid beta precursor protein (APP)
MCI: mild cognitive impairment
MMSE: Mini-Mental State Examination
MR: magnetic resonance
SNP: single-nucleotide polymorphism
SORL1: sortilin-related receptor 1
SORLA: sorting-related receptor with type-A repeats
VPS10P: vacuolar protein sorting 10 protein.
